# Current evidence on the role of lipid lowering drugs in the treatment of psoriasis

**DOI:** 10.3389/fmed.2022.900916

**Published:** 2022-08-11

**Authors:** Jiao Wang, Shuo Zhang, Meng Xing, Seokgyeong Hong, Liu Liu, Xiao-Jie Ding, Xiao-ying Sun, Ying Luo, Chun-xiao Wang, Miao Zhang, Bin Li, Xin Li

**Affiliations:** ^1^Department of Dermatology, Yueyang Hospital of Integrated Traditional Chinese and Western Medicine, Shanghai University of Traditional Chinese Medicine, Shanghai, China; ^2^Institute of Dermatology, Shanghai Academy of Traditional Chinese Medicine, Shanghai, China; ^3^Department of Dermatology, Shaanxi Hospital of Traditional Chinese Medicine, Xi’an, China; ^4^Shanghai Skin Disease Hospital, School of Medicine, Tongji University, Shanghai, China

**Keywords:** lipid metabolism, lipid-lowering drugs, statins, systematic review, meta-analysis, psoriasis

## Abstract

**Background:**

Abnormal lipid distribution is observed in patients with psoriasis, which increases their risk for atherosclerosis. Lipid-lowering drugs have a certain curative effect in the treatment of psoriasis, but there is no relevant evidence-based medical evaluation.

**Objective:**

The purpose of this systematic evaluation was to assess the efficacy, safety, and potential mechanisms of action of lipid-lowering drugs for the treatment of psoriasis.

**Methods:**

The PubMed, Embase, Cochrane Central Register of Controlled Trials, Clinical Trial, Chinese National Knowledge Infrastructure (CNKI), China Science and Technology Journal Database, and Wanfang Database were searched for relevant articles from inception to 31 December 2021. The RevMan 5.3 and Cochrane risk-of-bias tool were used for data analysis and risk assessment, respectively. The psoriasis area and severity index (PASI) score is the primary outcome indicator in clinical studies. Based on preclinical studies, we elucidated and mapped the action mechanisms of lipid-lowering drugs in the treatment of psoriasis.

**Results:**

The study included eight randomized controlled studies, four single-arm studies, and four *in vitro* studies. The results showed that lipid-lowering drugs, particularly statins, administered both orally and topically, can significantly improve psoriatic skin lesions and reduce the PASI scores [standardized mean difference, (SMD): −0.94; 95% CI: [−1.58, −0.31]; *p* = 0.004]. Oral statins performed best at week eight (SMD: −0.92; 95% CI: [−1.39, −0.44]; *p* = 0.0001). The mechanism of lipid-lowering drugs in the treatment of psoriasis may be related to the inhibition of keratinocyte proliferation, inhibition of CCL20–CCR6 interaction, and reduction in the levels of inflammatory factors.

**Limitations:**

There are few studies on lipid-lowering drugs and psoriasis, and their small sample sizes may render the evidence unconvincing.

**Conclusion:**

The present findings suggest that lipid-lowering drugs are relieving symptoms in psoriasis. Lipid-lowering drugs, particularly statins, can be used to treat psoriasis with good efficacy and few side effects.

## Introduction

Psoriasis is a chronic, recurrent, inflammatory, and immune-mediated systemic disease induced by the interaction between genetics and the environment (1), which is characterized by erythema covered with silvery white scales with punctate hemorrhages. Naïve T cells and keratinocytes (KC) activated by antigen-presenting cells, ultimately leading to the hyperproliferation of KC and accompanying inflammation (2). The incidence of psoriasis is increasing annually. In Australia, the prevalence of psoriasis has increased from 0.14 to 1.99% (3). In the United Kingdom, it is reported to be 1.5–2.8%, whereas in China, it is 0.47% (4). Psoriasis is not only a skin disease but also a systemic disease, which can co-exist with metabolic diseases such as diabetes (5), obesity (6), metabolic syndrome (7), and atherosclerosis (8). The pathogenesis of psoriasis has been extensively studied in terms of inflammation and immunosuppression. However, the exact mechanism is still unclear. The most widely studied mechanism involves the interleukin-23 (IL-23)/T-helper 17 (Th 17) immune axis (9). The levels of IL-23 and Th17-related cytokines, such as IL-17, IL-6, and IL-22, are elevated in patients with psoriasis compared to normal people. IL-23 is a key cytokine involved in the pathogenesis of psoriasis. The IL-23 signaling heterodimeric complex, composed of IL-23R and IL-12Rβ1, can activate transcriptional activators 3 (STAT3) to further lead to inflammation (10). On the other hand, keratin 16 (K16) also promotes massive proliferation of KC, and one study showed that IL-23 increases the expression of K16 (11). Modern medicine uses various therapies such as external medication, systemic therapy, and phototherapy, which are better for mild psoriasis, but less meaningful for severe psoriasis. In recent years, newly developed biological agents such as IL-23 inhibitors and TNF-α inhibitors seem to have brought new hope to patients with severe psoriasis, but the high treatment cost or some adverse reactions limit the widespread use of these drug (12). Thus, a proactive approach to psoriasis treatment is imperative.

Psoriasis is reported to be closely related to lipid metabolism, and patients with psoriasis have abnormal plasma lipid levels lately (13). Specifically, patients with psoriasis have elevated levels of low-density lipoprotein (LDL), triglycerides (TG), and total cholesterol (TC), but lower levels of high-density lipoprotein (HDL), and very low-density lipoprotein (VLDL) compared to normal individuals (14, 15). Recent studies have revealed that lipid-lowering drugs can help manage blood lipid levels in patients with psoriasis and reduce their cardiovascular risk while also treating psoriatic skin lesions (16). Lipid-lowering drugs primarily include statins, fibrates, niacin, bile acid chelators, and proprotein convertase subtilisin kexin 9 (PCSK9) inhibitors (17). Due to the high cholesterol-lowering function, statins have been used in the prevention and treatment of cardiovascular diseases. In recent years, the independent lipid-lowering effect of statins has become a hot topic in clinical practice. In addition, statins, the inhibitors of hydroxymethylglutaryl coenzyme A (HMG-CoA) reductase, have various pharmacological effects, such as anti-inflammatory effects by inhibiting antigen cell presentation and lymphocyte activation. High-dose simvastatin can also inhibit vascular proliferation by mediating vascular endothelial growth factor (VEGF), which is the typical pathological feature of psoriasis. Statin also has anti-oxidative stress, anti-thrombotic and other effects (18).

In patients with hyperlipidemia, statins can inhibit HMG-CoA reductase, the rate-limiting enzyme of cholesterol synthase, stimulating the cellular synthesis of LDL receptors (LDL-R), which increases the number and activity of LDL-R on the membrane surface of hepatocytes. LDL-R can accelerate LDL-c clearance from serum, eventually causing a decrease in serum TC levels (19). Weitz-Schmidt et al. (20) found that statins can inhibit β2 integrin leukocyte function antigen-1 (LFA-1), which is involved in lymphocyte circulation and leukocyte extravasation, by binding to unknown integrin sites that play an important role in inflammation. Krueger et al. found that anti-LFA-1 antibodies inhibit cutaneous T-cell trafficking, thereby improving the symptoms of psoriasis (21). Thus, the selective inhibition of an unknown allosteric site in LFA-1 can be used to treat psoriasis. It is well known that TNF-α is positively correlated with psoriasis. Statins have been reported to significantly reduce TNF-α levels (22, 23), opening a new path in psoriasis treatment.

Fibrates exert lipid-lowering effects by reducing TG levels and increasing high-density lipoprotein cholesterol (HDL-C) levels, and exert indirect anti-inflammatory effects by reducing the levels of adhesion molecules through the above pathways (24). Fibrates can also reduce the levels of pro-inflammatory factors such as IL-6, IL-1β, and TNF-α to exert direct anti-inflammatory effects (25). Combination of statin and niacin can significantly increase high-density lipoprotein levels and reduce LDL levels, which are protective factors for cardiovascular events. Studies have shown that monomethyl fumarate, as an agonist of the niacin receptor GPR109A, has antioxidant, anti-inflammatory and immunomodulatory effects for inflammatory diseases such as psoriasis. Therefore, niacin may become a new target for the treatment of psoriasis (26). PCSK9 is a serine protease. After combining with low density lipoprotein receptors, it will accelerate the explanation, thereby raising the LDL-C level in the plasma. The PCSK9 inhibitor reverses this way. Not only that, it can also reduce the level of lipoprotein (A) in plasma, which is not available by his diced drugs (27).

Although psoriasis is associated with a high risk for lipid metabolism disease, it is uncertain whether treating patients for lipid metabolism disease will improve their condition of psoriasis. In addition, the efficacy of lipid-lowering drugs in the treatment of psoriasis still lacks strong evidence, and the underlying mechanisms have not yet been fully elucidated. Therefore, by reviewing the relevant literature, including clinical and preclinical studies involving lipid-lowering drugs, we aimed to determine the safety and efficacy of lipid-lowering drugs in the treatment of psoriasis and to elucidate their action mechanism.

## Methods and analysis

### Search strategy

The following databases were searched for literature on the use of lipid-lowering drugs in the treatment of psoriasis, from inception to 31 December 2021: PubMed, Embase, Cochrane Central Register of Controlled Trials, Clinical Trial, Chinese National Knowledge Infrastructure (CNKI), China Science and Technology Journal Database, and Wanfang Database. Through the PICOS principle, we found the subject headings and free words in the Medical Subject Headings (MeSH) in the PubMed database and retrieved relevant literature using a combination of these subject headings and free words. We divided the search terms into two categories. One was for psoriasis, including “Psoriasis,” “Psoriases,” “Pustulosis of Palms and Soles,” “Pustulosis Palmaris et Plantaris,” “Palmoplantaris Pustulosis,” and “Pustular Psoriasis of Palms and Soles.” Another search was done for lipid-lowering drugs, including the terms “Statins,” “Fibrates,” “Niacin,” “Ezetimibe,” “PCSK9 inhibitors,” and “bile acid sequestrants.” To be more comprehensive, we also searched for “lipid metabolism,” “hyperlipidemia,” “hypercholesterolemia,” “hyperlipidemia,” “hyperlipidemia,” “hyperlipoproteinemia,” and “psoriasis.”

### Outcomes

The psoriasis area and severity index (PASI) is used as the primary outcome measure in this study. The dermatology life quality index (DLQI) score, LDL-C, adverse events (AE), IL-17, IL-8, TNF-α, and vascular cell adhesion molecule 1 (VCAM-1) were used as secondary outcome measures. We elucidated the mechanism of action of lipid-lowering drugs in the treatment of psoriasis by summarizing and analyzing the changes in cell phenotype and function after lipid-lowering drug intervention.

### Inclusion criteria

Studies that met the following criteria were included in this systematic review: (1) randomized controlled trials and single-arm trials of lipid-lowering drugs in the treatment of psoriasis or psoriasis co-morbidities, (2) animal or cell models of interventions involving lipid-lowering drugs, and (3) psoriasis models. The exclusion criteria were as follows: (1) case reports and observational studies, (2) reviews and conferences, (3) publications with insufficient basic information, such as baseline data in patients with psoriasis, (4) duplicate publications, and (5) publications whose full text cannot be found.

### Data extraction

The two authors (LL and SZ) screened the literature by reading the titles, abstracts, and full texts and independently extracted the relevant information. The clinical study information gathered included (1) first author, (2) year of publication, (3) type of psoriasis, (4) sample size, (5) sex, (6) age, (7) intervention, and (8) duration of treatment. For preclinical studies, the information collected were (1) *in vitro* study, (2) interventions, (3) outcome measures, (4) main findings, and (5) signaling pathways involved. Data are presented as the mean ± SD. If disagreements arose, another author (JW) intervened and negotiated between LL and SZ to reach an agreement.

### Risk-of-bias assessment

Two reviewers (C-XW and MZ) used the Cochrane risk-of-bias tool to independently assess the risk-of-bias in the included studies. The parameters assessed were as follows: random sequence generation, allocation concealment, blinding of participants and personnel, blinding of the outcome assessment, incomplete outcome data, selective reporting, and other biases. The results were classified as low risk, high risk, or unclear. In case of disagreement, a third author (JW) joined the discussion to reach an agreement.

### Statistical analysis

Meta-analyses were performed using RevMan 5.3 and Stata software provided by the Cochrane Collaboration. Continuous data were expressed as the mean difference (MD) and 95% confidence interval (CI). The *I*^2^ statistic test for heterogeneity was also performed. If *p* > 0.1 and *I*^2^ < 50%, the results were homogeneous, and the fixed effect model was used; however, if *p* > 0.1 and *I*^2^ > 50%, there was heterogeneity in the results, and a random-effects model was used. Subgroup analysis was then performed to avoid heterogeneity.

## Results

### Selection and characteristics of studies

**FIGURE 1:**
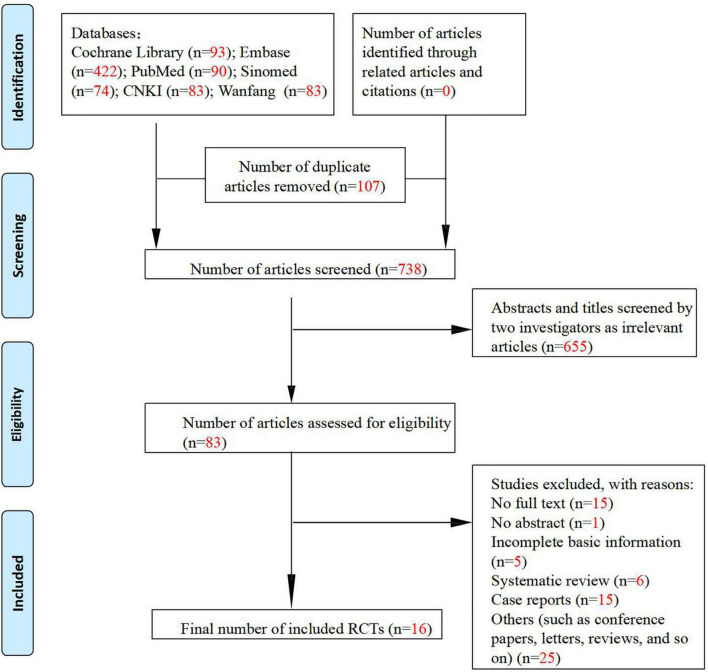
Flowchart of study inclusion according to the PRISMA 2009 guidelines.

A total of 1,663 articles were retrieved from the seven databases searched, and 738 articles were left after removing duplicate publications, among which 655 irrelevant articles were further excluded. The full texts of the remaining 83 articles were reviewed to exclude articles with incomplete basic information, as well as reviews and case reports. Finally, 16 articles were included in this meta-analysis, including eight randomized controlled clinical studies (28–35), four single-arm clinical studies (Figure 1) (36–39), and four preclinical studies (40–43). The characteristics of the included clinical and preclinical studies are presented in Supplementary Tables 1, 2, 3. One clinical study (31) administered tetradecylthioacetic acid (TTA) at an oral dose of 1,000 mg (five capsules) as the intervention. The other seven studies administered statins, including simvastatin (28, 29, 33–35) and atorvastatin (30, 32). One study (29) administered statins topically, and the rest administered statins at an oral dose of 40 mg per day. Three clinical studies (28, 32, 34) did not mention the type of psoriasis, while the remaining studies were on plaque psoriasis. Notably, there is a lack of data from the control group after LDL-c treatment in one randomized controlled study (34), and therefore, we classified this study as a single-arm study. All preclinical studies included in the meta-analysis were *in vitro* experiments using three cell lines: human keratinocytes, CD4+ T cells, and HaCaT cells. The intervention used in one study (40) was tauroursodeoxycholic acid (TUDC), and the remaining three studies administered statins.

### Risk-of-bias

Overall, the quality of the studies included in the meta-analysis was high, and all studies used randomized grouping; however, the specific randomization methods were not clearly stated, leaving insufficient information to determine whether they were low-risk. Three studies (29, 32, 34) used a blinded approach.

**FIGURE 2:**
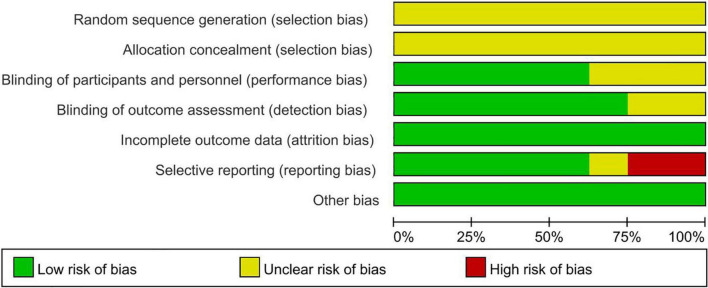
Risk of bias graph of clinical studies.

**FIGURE 3:**
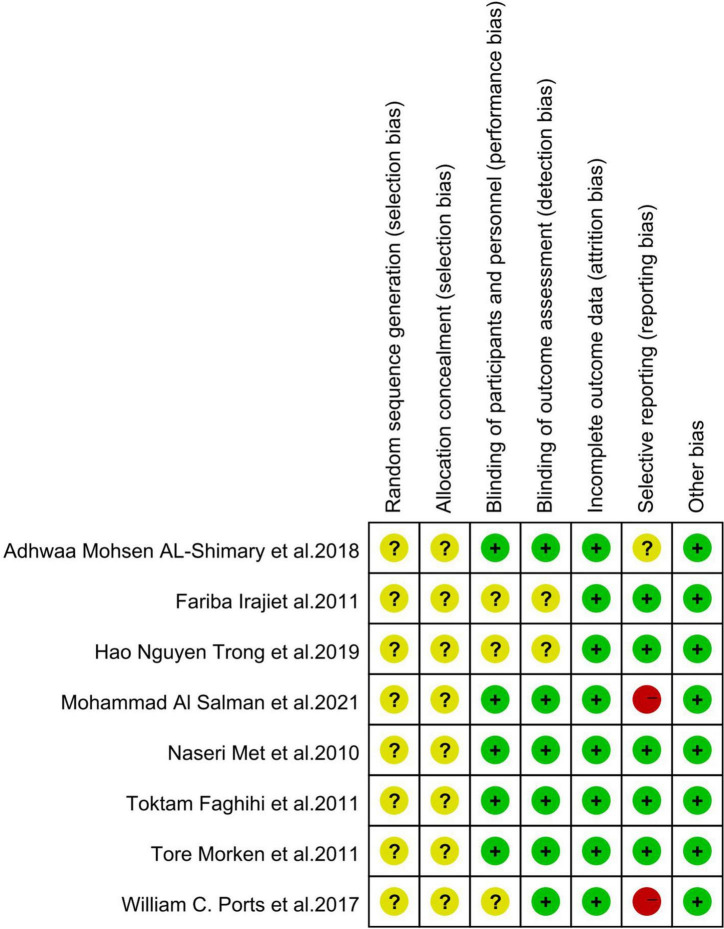
Risk of bias summary of clinical studies.

Eight studies reported detailed outcome metrics, indicating a low risk for attrition bias. One study (33) did not report a pre-stated indicator; however, it was not a primary outcome indicator; it had little impact on the study’s outcome, and therefore, we classified it as unclear risk. Two studies (32, 34) reported incomplete outcome indicators and missing data and were classified as high risk. Risk of bias is presented in Figure 2 and Figure 3.

### Outcomes of clinical studies

#### Drug therapy

##### Statin therapy

A meta-analysis of six studies (28–30, 33–35) evaluated the PASI scores after statin treatment for psoriasis. As expected, patients with psoriasis experienced a decrease in PASI scores after treatment with oral (SMD: −0.47; 95% CI: [−0.90, −0.03]; *p* = 0.03) or topical statins (SMD: −1.91; 95% CI: [−3.58, −0.24]; *p* = 0.03). However, because of the large heterogeneity in the results of the included studies, we divided the patients into three subgroups of different treatment durations. In the fourth week, oral statins were ineffective (SMD: −0.14; 95% CI: [0.65, −0.37]; *p* = 0.59), whereas topical statin creams were effective (SMD: −4.13; 95% CI: [−4.97, −3.29]; *p* < 0.00001). In the eighth week, both oral and topical statins were effective (oral statins: SMD: −0.92; 95% CI: [−1.39, −0.44]; *p* = 0.0001; topical statins SMD: −0.94; 95% CI: [−1.43, −0.45]; *p* = 0.0002). When the treatment time reached 12 weeks, the oral statins were ineffective (SMD: 0.05; 95% CI: [−0.39, −0.49]; *p* = 0.82), whereas topical statins were still effective (SMD: 0.78; 95% CI: [−1.26, −0.29]; *p* = 0.002) (Table 1). The random-effects model meta-analysis of three single-arm studies (36, 37, 39) showed that statins reduced PASI scores by 9.26% in patients with psoriasis (95% CI: [7.29, 11.23]) (Table 2). A randomized controlled study (35) reported that statins significantly improved DLQI in patients with psoriasis. Similarly, two single-arm studies (36, 39) revealed that statins reduced DLQI by 12.30% (95% CI: [3.77, 20.82]) (Table 3), and two single-arm studies (34, 39) revealed that statins reduced LDL-c levels by 28.37% (95% CI: [3.01, 53.72]) in patients with psoriasis (Table 4). Only one randomized controlled study (35) reported adverse reactions, such as nausea, headache, dyspepsia, increased appetite, muscle weakness, and insomnia, in patients with psoriasis treated with statins. A single-arm study (36) reported adverse effects such as severe headache, arterial hypertension, and a mild elevation of transaminase levels. A single-arm study primarily reported myalgia as an AE. A randomized controlled study (39) reported significant reductions in IL-17 and TNF-α levels in patients with psoriasis after taking statins for eight weeks.

**TABLE 1:** Subgroup analysis of PASI scores in randomized controlled study.

References	Comparison	Change from baseline (Mean ± SD)		Standardized mean difference IV random 95% CI	*P*-Value
		E	C		
**1. PASI (4W)**					
**1.1 Oral**					
Trong et al. (34) (a)	Oral simvastatin 40 mg, bid + topical calcipotriol/betamethasone dipropionate ointment vs. topical calcipotriol/betamethasone dipropionate ointment	8.58 ± 5.62	9.34 ± 5.01	−0.14 [−0.65, 0.37]	
Subtotal (95% CI)				−0.14 [−0.65, 0.37]	*p* = 0.59
**1.2 Topical**					
Iraji et al. (63) (a)	Topical simvastatin 3% ointment, bid + calcipotriol 0.005% ointment, bid vs. calcipotriol 0.005% ointment, bid	2.49 ± 0.22	3.54 ± 0.28	−4.13 [−4.97, −3.29]	
Subtotal (95% CI)				−4.13 [−4.97, −3.29]	*p* < 0.00001
Total (95% CI) *I*^2^ = 98%				−2.12 [−6.03, 1.79]	*p* = 0.29
**2. PASI (8W)**					
**2.1 Oral**					
Naseri et al. (28)	Simvastatin + topical steroid vs. placebo tablets + topical steroid	3.83 ± 0.11	3.98 ± 0.11	−1.33 [−2.13, −0.53]	
Trong et al. (34) (b)	Oral simvastatin 40 mg, bid + topical calcipotriol/betamethasone dipropionate ointment vs. Topical calcipotriol/betamethasone dipropionate ointment	4.17 ± 3.81	6.52 ± 4.89	−0.53 [−1.04, −0.01]	
AL-Shimary et al. (33)	Oral simvastatin 40 mg, qd + topical steroids vs. betamethasone dipropionate 0.5 mg/day and salicylic acid 30 mg/day	3.85 ± 2.34	7.87 ± 4.60	−1.08 [−1.68, −0.49]	
Subtotal (95% CI) *I*^2^ = 42%				−0.92 [−1.39, −0.44]	*p* = 0.0001
**2.2 Topical**					
Iraji et al. (63) (b)	Topical simvastatin 3% ointment, bid + calcipotriol 0.005% ointment, bid vs. calcipotriol 0.005% ointment, bid	1.87 ± 0.20	2.1 ± 0.28	−0.94 [−1.43, −0.45]	
Subtotal (95% CI)				−0.94 [−1.43, −0.45]	*p* = 0.0002
Total (95% CI) *I*^2^ = 13%				−0.90 [−1.21, −0.59]	*p* < 0.00001
**3. PASI (12W)**					
**3.1 Oral**					
Al Salman et al. (35)	Oral simvastatin 40 mg, qd + NB−UVB 3 times/week vs. oral placebo 40 mg, qd + NB−UVB 3 times/week	4.18 ± 3.45	4.36 ± 3.70	−0.05 [−0.67, 0.57]	
Faghihi et al. (30)	Oral atorvastatin 40 mg, qd + emollients, keratolytics, and/or class corticosteroids vs. oral placebo + emollients, keratolytics, and/or class corticosteroids	2.94 ± 2.37	2.59 ± 2.09	0.15 [−0.47, 0.77]	
Subtotal (95% CI) *I*^2^ = 0%				0.05 [−0.39, 0.49]	*p* = 0.82
**3.2 Topical**					
Iraji et al. (63) (c)	Topical simvastatin 3% ointment, bid + calcipotriol 0.005% ointment, bid vs. calcipotriol 0.005% ointment, bid	1.55 ± 0.19	1.72 ± 0.24	−0.78 [−1.26, −0.29]	
Subtotal (95% CI)				−0.78 [−1.26, −0.29]	*p* = 0.002
Total (95% CI) *I*^2^ = 69%				−0.25 [−0.84, 0.34]	*p* = 0.40

**TABLE 2:** Meta analysis of PASI scores in single arm study.

References	Intervention method	Effect (95% CI)	Weight (%)
Asad et al. (39)	Atorvastatin 40 mg tid for the first 3 m followed by 20 mg/day for the next 3 m+ topical betamethasone valerate 0.1% qd for 6 m	9.54 [9.43, 9.65]	35.72
Shirinsky et al. (64)	Simvastatin 40 mg/day	11.50 [9.91, 13.09]	28.98
Aslam et al. (37)	Simvastatin 40 mg/day	7.14 [6.77, 7.51]	35.30
Overall, DL, random-effects (*I*^2^ = 98.7%, *p* = 0.000)		9.26 [7.29, 11.23]	100.00

**TABLE 3:** Meta analysis of DLQI scores in single arm study.

References	Intervention method	Effect (95% CI)	Weight (%)
Asad et al. (39)	Atorvastatin 40 mg tid for the first 3 m followed by 20 mg/day for the next 3 m+ topical betamethasone valerate 0.1% qd for 6 m	16.63 [16.48, 16.78]	50.17
Shirinsky et al. (64)	Simvastatin 40 mg/day	7.93 [6.92, 8.94]	49.83
Overall, DL, random-effects (*I*^2^ = 98.6%, *p* = 0.000)		12.30 [3.77, 20.82]	100.00

**TABLE 4:** Meta analysis of LDL-c in single arm study.

References	Intervention method	Effect (95% CI)	Weight (%)
Asad et al. (39)	Atorvastatin 40 mg tid for the first 3 m followed by 20 mg/day for the next 3 m+ topical betamethasone valerate 0.1% qd for 6 m	15.43 [15.39, 15.47]	50.00
Chodick et al. (38)	Stains	41.30 [41.27, 41.33]	50.00
Overall, DL, random-effects (*I*^2^ = 100.0%, *p* = 0.000)		28.37 [3.01, 53.72]	100.00

##### Fish oil therapy

Our team included 18 randomized controlled studies and conducted a meta-analysis of fish oil and its main constituent omega-3 PUFAs for psoriasis management, demonstrating that fish oil as monotherapy did not improve psoriasis, but when combined with conventional treatment, it can improve the PASI score and lesion area in patients with psoriasis, and also have a certain alleviation effect on itching. Not only that, it has no side effects and can be used as a comprehensive management method for psoriasis and its comorbidities (44). A nearly 2-year stacked double-blind placebo-controlled study showed that taking herring roe oil for 26 weeks improved PASI scores in plaque psoriasis (45).

##### Other lipid-lowering drugs

Besides statins and fish oil, there are other types of lipid-lowering drugs, such as fibrates, bile acid sequestrants, ezetimibe, niacin, and PCSK9 inhibitors. However, there have been no clinical studies on their use in psoriasis. TTA is a 3-thio fatty acid with biologically active hypolipidemic effects. A pilot study (31) reported that treating psoriasis with TTA resulted in significant reductions in the levels of triglycerides and total fatty acids, total plasma cholesterol, LDL/HDL cholesterol ratio, and non-HDL cholesterol after four weeks of treatment. In addition, TTA treatment also led to reductions in plasma levels of TNF-α, VCAM-1, and IL-8.

## Discussion

To the best of our knowledge, this is the first comprehensive systematic evaluation and meta-analysis of the clinical efficacy and related mechanisms of lipid-lowering drugs for the treatment of psoriasis. This review primarily included clinical and preclinical studies. We used the PASI score as the primary efficacy index for the clinical studies, with DLQI, LDL-c, AEs, IL-17, IL-8, TNF-α, and VCAM-1 as the secondary outcome indices. Based on the results reported from the preclinical studies, we elucidated the action mechanism of lipid-lowering drugs in the treatment of psoriasis. Hence, we demonstrated that lipid-lowering drugs are an important protective factor for psoriasis along with CVD. Psoriasis is a chronic inflammatory disease, and inflammation and infection can cause changes in lipid levels (46). Nowowiejska et al. (47) believe that psoriasis is closely related to lipid abnormalities. Specifically, lipid expression and metabolism are abnormal in psoriasis patients, mainly manifested in increased LDL receptor and lipoprotein-related receptor, oxidized modified lipoproteins (ox-LDL) and lectin-type ox-LDL receptor. Lectin-type ox-LDL receptor 1 (LOX-1) is also involved in the expression of IL-23, a key inflammatory factor in the pathogenesis of psoriasis. In terms of lipid metabolism, ceramides, which are closely related to the skin barrier, and free fatty acids that provide energy to tissues, were significantly reduced in psoriatic skin lesions. Likewise, patients with psoriasis have abnormal blood lipid profiles, elevated serum concentrations of most adipokines, and abnormal fatty acid-binding proteins. Therefore, lipid-lowering drugs can alleviate psoriasis dyslipidemia and relieve skin lesions through different action pathways.

**FIGURE 4:**
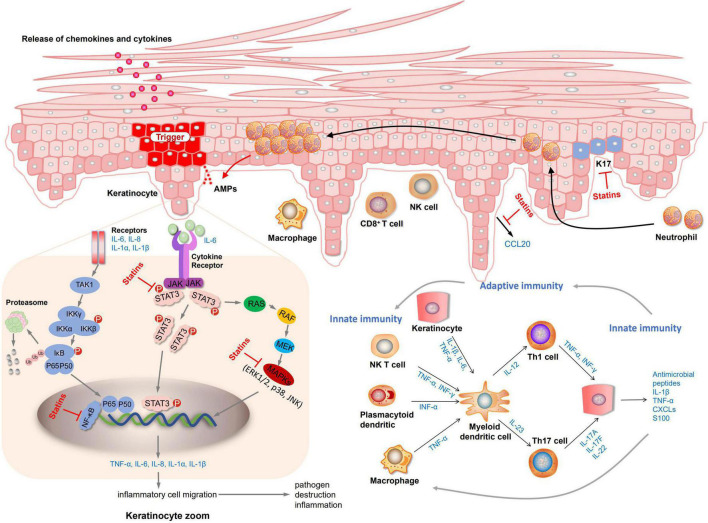
Diagram of the mechanism of preclinical in vitro.

Elevated LDL-c levels have been shown to be a risk factor for CVD (48, 49). The latest National Cholesterol Education Program (NCEP) guidelines state that patients at high risk of coronary heart disease would better get their cholesterol level below 100 mg/dL (50). Joint AAD-NPF guidelines also recommend regular lipid screening in patients with moderate to severe psoriasis (13). Nevertheless, is it safe and effective to treat psoriasis by taking lipid-lowering drugs to improve hyperlipidemia further? There is not enough evidence yet. A systematic review of three randomized controlled studies showed that oral statins can significantly reduce the severity of psoriasis skin disease (51). Statins are the most widely studied lipid-lowering drugs. Our study demonstrated that statins could reduce LDL-c levels and lower PASI scores in patients with psoriasis, providing a new approach for treating psoriasis. An actual meta-analysis of 737 patients showed that statin therapy significantly improved the disease activity score (DAS28), tender joint count, erythrocyte sedimentation rates (ESR), and C-reactive protein (CRP) in patients with rheumatoid arthritis (RA) relative to placebo (52). Another meta-analysis including 15 studies involving 992 patients also confirmed the above results (53). As a consequence, psoriasis may can achieve good curative effects through statin treatment as an inflammatory disease. Further exploration through preclinical studies has demonstrated that statins treat psoriasis by reducing the levels of inflammatory factors. Both mevastatin and atorvastatin can inhibit NF-κB activation and inhibit the release of inflammatory factors TNF-α or chemokines (42, 43). Mevastatin can also inhibit MAPKs (JNK, p38, ERK 1/2) and STAT3 signaling pathways, inhibit the expression of keratin and inflammatory factors (43). Fluvastatin and simvastatin prevent inflammation by inhibiting the release of the elevated chemokine CCL20 in psoriasis and further inhibiting the interaction between CCL20 and CCR6 (41). The mechanism diagram of lipid-lowering drugs in the treatment of psoriasis is shown in Figure 4. Yet there are few preclinical studies on statins at present, and it is unclear whether all type of statins can exert anti-inflammatory, inhibit keratinocyte proliferation, inhibit the release of chemokines to exert therapeutic effects.

Fibrates are peroxisome proliferator-activated receptor alpha (PPARα) agonists that regulate lipid metabolism and reduce inflammation through transcriptional regulation. A study of 27 patients with RA taking conventional medication and fenofibrate for 3 months showed fibrate treatment reduced the disease activity score (DAS28) and CRP and IL-6 levels (54). Similarly, ezetimibe, a lipid-lowering drug, also has anti-inflammatory effects. Maki-Petaja et al. demonstrated that ezetimibe reduced the levels of inflammatory markers, erythrocyte sedimentation rate (ESR), and CRP in patients RA (55). No studies have been conducted on the effect of bile acid binders on inflammatory diseases. Although there are no clinical studies related to these three lipid-lowering drugs for the treatment of psoriasis, we believe that these drugs will also play a therapeutic role in psoriasis.

Another promising lipid-lowering drug is PCSK9. PCSK9 is a neuroapoptosis-regulating convertase that helps in liver regeneration, regulates neuroapoptosis, and affects LDL internalization by decreasing the amount of LDL-R in hepatocytes. This prevents LDL clearance from the blood leading to hypercholesterolemia. Studies have revealed that PCSK9 levels are significantly associated with cholesterol, ox-LDL, and triglyceride levels (56). The results of four randomized, double-blind placebo trials (57–59), demonstrated that PCSK9 inhibitors significantly lowered LDL-c levels, reduced the incidence of CVD. Further, PCSK9 inhibitors are more potent than statin-based drugs. In terms of safety, the only adverse effects were injection site reactions (57), without the risk of transaminase elevation and muscle pain that may be associated with statin drugs (60). For most people, lipid control can be achieved with moderate doses of statins. However, some patients with familial hypercholesterolemia typically experience a more severe elevation of LDL-c levels; hence, lipid control cannot be achieved by taking statins alone. Consequently, clinicians can use PCSK9 inhibitors alone or in combination with statins to manage dyslipidemia. Studies have revealed that a combination of PCSK9 inhibitors and statins can reduce LDL-c levels by 59–60% compared to statins alone (61). Nevertheless, there are no clinical studies on the effect of PCSK9 on psoriasis. Based on its efficacy, safety profile, and negligible adverse effects, PCSK9 could be a new option in the treatment of psoriasis in combination with lipid metabolic disorders.

Our previous studies have found that the bioactive lipid sphingosine-1-phosphate (S1P) produced by the sphingolipid metabolite ceramidase plays an important role in autoimmune diseases, and S1P inhibitors can improve psoriasis by reducing the number of lymphocytes and inflammatory factors (1). A recent study showed that antagonists of cannabinoids and eicosanoids, the products of enzymatic lipid metabolism, suppressed the inflammatory response in an animal model of psoriasis. It also has the potential to be a new way to treat psoriasis (62).

Clinical studies on lipid-lowering drugs and psoriasis are scarce and involve a small number of patients, and their evidence may not be sufficient to demonstrate the efficacy and safety of lipid-lowering drugs as a treatment for psoriasis. However, based on the current systematic evaluations and meta-analyses, we have demonstrated the clinical utility of lipid-lowering drugs, particularly statins, for the treatment of psoriasis.

In addition, the mechanisms of lipid-lowering drugs for psoriasis have not been comprehensively studied, and only one article in our included studies has elaborated on these mechanisms. However, the mechanisms of action of lipid-lowering drugs in psoriasis based on new preclinical evidence as evaluated in this review may provide new insights into this association. Therefore, large-scale, high-quality controlled trials and preclinical studies are required to confirm the efficacy and molecular mechanisms of lipid-lowering drugs in treating psoriasis.

## Conclusion

Our findings suggest that lipid-lowering drugs, particularly statins, can significantly improve psoriasis skin lesions and reduce PASI scores. The mechanism of lipid-lowering drugs in the treatment of psoriasis may be related to the inhibition of keratinocyte proliferation, inhibition of CCL20–CCR6 interaction, and reduction in the levels of inflammatory factors.

## Data availability statement

The original contributions presented in this study are included in the article/Supplementary material, further inquiries can be directed to the corresponding authors.

## Author contributions

XL and BL proposed and designed the study. XL obtained the funding support and revised the manuscript. JW, LL, SZ, and X-YS retrieved and selected the studies. C-XW and MZ extracted the data. YL and X-JD assessed the quality of all studies. LL, JW, and X-YS performed the statistical analyses of all data. JW drafted the manuscript. All authors contributed to the article and approved the submitted version.
