# Supplementary material for: Current evidence on the role of lipid lowering drugs in the treatment of psoriasis
Source: Front Med (Lausanne). 2022 Aug 11;9:900916. doi: 10.3389/fmed.2022.900916 (PMC9403729; doi:10.3389/fmed.2022.900916)
Supplement: Supplementary file 1 [file Table_1.DOCX]

| **Table S1. Characteristics of the included randomized controlled studies.** | | | | | | | | | | | |
| --- | --- | --- | --- | --- | --- | --- | --- | --- | --- | --- | --- |
| **Author Year** | **Sample Size** | | **Average Age (years) (Mean±SD)** | | **Gender(M/F)** | | **Types of Psoriasis** | **Intervention** | | **Course of  Treatment** | **Outcome** |
|  | **E** | **C** | **E** | **C** | **E** | **C** |  | **E** | **C** |  |  |
| Naseri Met  et al. 2010 | 15 | 15 | 38.5 | 45.5 | 9/6 | 11/4 | N/A | oral **simvastatin** 20 mg, bid + topical steroid (50% betamethasone in petrolatum) | oral placebo + the same topical steroid | 8 weeks | PASI |
| Fariba Iraji  et al. 2011 | 40 | 40 | 32.5±10.7 | 31.55±9.5 | 18/22 | 21/19 | Plaque Psoriasis | Topical **simvastatin** 3% ointment**,** bid + calcipotriol 0.005% ointment, bid | calcipotriol 0.005% ointment bid | 12 weks | PASI |
| Toktam Faghihi  et al. 2011 | 20 | 20 | 43.85±14.32 | 36.55±12.11 | 8/12 | 12/8 | Plaque Psoriasis | oral **atorvastatin** 40 mg, qd + emollients, keratolytics, and/or class corticosteroids | oral placebo + emollients, keratolytics, and/or class corticosteroids | 12 weeks | PASI, BSA |
| Tore Morken  et al. 2011 | 21 | 22 | 41.4 | 44.4 | 12/9 | 8/14 | Plaque Psoriasis | oral **TTA** 1000 mg (5 capsules), qd | oral placebo 1000 mg, qd | 4 weeks | TC, Non-HDL-c, LDL-c,  HDL-c/LDL-c, TG, TFA, VCAM-1, IL-8, TNF-a |
| William C. Ports et al. 2017 | 21 | 31 | N/A | N/A | N/A | N/A | N/A | oral **atorvastatin** 10 mg, qd | oral placebo 10 mg, qd | 186 weeks | TC, TG, LDC-c,  HDC-c, TC:HDL-c |
| Adhwaa Mohsen AL-Shimary  et al. 2018 | 25 | 25 | N/A | N/A | N/A | N/A | Plaque Psoriasis | oral **simvastatin** 40 mg, qd + topical steroids | betamethasone dipropionate 0.5 mg/d and salicylic acid 30 mg/d | 8 weeks | PASI, IL-17, TNF-a |
| Hao Nguyen Trong  et al. 2019 | 30 | 30 | 36.0±10.0 | 39.1±14.5 | 17/13 | 17/13 | N/A | oral **simvastatin** 40 mg, bid + topical calcipotriol/betamethasone dipropionate ointment | Topical calcipotriol/betamethasone dipropionate ointment | 8 weeks | PASI, TC, TG,  LDC-c, HDC-c |
| Mohammad Al Salman  et al. 2021 | 20 | 20 | 41 | 46.9 | 10/10 | 12/8 | Plaque Psoriasis | oral **simvastatin** 40 mg, qd + NB-UVB 3 times/week | oral plecebo 40 mg, qd + NB-UVB  3 times/week | 12 weeks | PASI, DLQI |

**Abbreviations:** TTA, tetradecylthioacetic acid; TC, cholesterol; HDL, high-density lipoprotein; Non-HDL-C, free cholesterol HDL cholesterol; LDL, low-density lipoprotein; TG, triacylglycerol; TFA, total fatty acids; VCAM, vascular cell adhesion molecule; IL, interleukin; NB-UVB, narrow band-ultraviolet; VAS, Visual Analog Scales; PASI, psoriasis area and severity index; BSA, body surface area; DLQI, Dermatology Life Quality Index; N/A, Not applicable; M, Male; F, Female; w, weeks; m, months; y, years; bid, twice daily; qd, once a day; E, experiment; C, control.
