# Supplementary material for: Current evidence on the role of lipid lowering drugs in the treatment of psoriasis
Source: Front Med (Lausanne). 2022 Aug 11;9:900916. doi: 10.3389/fmed.2022.900916 (PMC9403729; doi:10.3389/fmed.2022.900916)
Supplement: Supplementary file 2 [file Table_2.DOCX]

| **Table S2. Characteristics of the included single arm studies.** | | | | | | | |
| --- | --- | --- | --- | --- | --- | --- | --- |
| **Author, Year** | **Sample Size** | **Average Age (years) (Mean±SD)** | **Gender (M/F)** | **Types of Psoriasis** | **Intervention** | **Course of  Treatment** | **Outcomes** |
| Ivan Valeryevich Shirinsky et al. 2007 | 7 | N/A | 3/4 | Plaque Psoriasis | Simvastatin 40 mg/d | 8 w | PASI |
| Shazia Aslam et al. 2013 | 60 | 35.47+12.39 | 40/20 | Plaque Psoriasis | Simvastatin 40 mg/d | 8 w | PASI, BSA, DLQI |
| G. Chodick et al. 2015 | 205820 | 12.16±4.20 | 94469/111351 | N/A | Statins | 6.2 y | LDL-c |
| Farah Asad et al. 2017 | 68 | 47.91±8.42 | 52/16 | Plaque Psoriasis | Atorvastatin 40 mg tid for the first 3m followed by 20 mg/d for the next 3m + topical betamethasone valerate 0.1% qd for 6m | 6 m | PASI, DLQI, hsCRP, LFTS, lipid profile |

**Abbreviations:** N/A, not applicable; PASI, psoriasis area and severity index; BSA, body surface area; DLQI, Dermatology Life Quality Index; M, male; F, female; w, weeks; m, months;

y, years; d, day; tid, three times daily; LDL, low-density lipoprotein; hsCRP, Highly sensitive C-reactive protein level; LFTs: liver function test.
