# Supplementary material for: Current evidence on the role of lipid lowering drugs in the treatment of psoriasis
Source: Front Med (Lausanne). 2022 Aug 11;9:900916. doi: 10.3389/fmed.2022.900916 (PMC9403729; doi:10.3389/fmed.2022.900916)
Supplement: Supplementary file 3 [file Table_3.DOCX]

| **Table S3. Characteristics of the included *in vitro* preclinical studies.** | | | | | |
| --- | --- | --- | --- | --- | --- |
| **Author, year** | **Cell Type** | **Intervention Methods** | **Cell Tracking Method** | **Outcomes** | **Pathway(s) Involved** |
| Yuji Yamaguchi  et al. 1998 | human keratinocytes | TUDC | BrdU: proliferation of keratinocytes | TUDC produces reversible growth-inhibitory effects on human keratinocytes via mechanisms other than cytotoxicity. | N/A |
| Tae-Gyun Kim et al. 2011 | CD4+ T cells | fluvastatin, simvastatin, pravastatin | ELISA: CCL20 | Fluvastatin and simvastatin significantly inhibited the production of CCL20 by HaCaT cells and inhibited the migration of CD4+ T cells to CCL20. | N/A |
| Nagaraj M  et al. 2014 | HaCaT | atorvastatin | ELISA: TNF-a | The inhibition of keratinocyte proliferation by atorvastatin may be due to the inhibition of cytokine release and NF-kB activation. | N/A |
| Min Young Kim et al. 2021 | HaCaT | Statins (atorvastatin, lovastatin, mevastatin, rosuvastatin, simvastatin)，Sigma Aldrich (St. Louis, MO, USA),  dissolved in dimethyl sulfoxide. | 1. RT-PCR: mRNA of IL-1α/β, IL-6, IL-8, CCL20  2. WB: P-ERK 1/2, P-JNK, P-p38, P-STAT3  3. RT-PCR: Keratin 5/14/6A/16  4. RT-PCR / WB: IL-6, CCL20, KRT 17, STAT3, P-STAT3 | 1. Mevastatin can inhibit psoriasis-induced cytokine and chemokine mRNA expression by regulating NF-κB signaling.  2. Mevastatin can inhibit the phosphorylation of MAPKs (ERK 1/2, JNK, and p38) and STAT3 and the transcriptional regulation of NF-κB in TNF-α signaling.  3. Mevastatin can significantly inhibit the expression of keratin, leading to hyperkeratinization and the overexpression of cytokines and chemokines in psoriatic skin.  4. Mevastatin can inhibit the expression of IL-6, CCL20, and KRT 17 by regulating IL-17A-induced STAT3 phosphorylation in human keratinocytes. | 1. IκB/NF-κB pathway  2. MAPKs (ERK 1/2, JNK, p38) and STAT3 pathway |

**Abbreviations**: TUDC, tauroursodeoxycholic acid; ELISA, enzyme-linked immunosorbent assay; RT-PCR, reverse transcription-polymerase chain reaction; WB, western blotting; IL, interleukin; KRT, keratin.
